# The effectiveness of respiratory training as a preventive strategy against cognitive decline: a mini review

**DOI:** 10.3389/fresc.2026.1778837

**Published:** 2026-03-09

**Authors:** Th. Zekis, E. Grammatopoulou, D. Tsimouris, V. Sakellari, I. Patsaki

**Affiliations:** Laboratory of Advanced Physiotherapy, Department of Physiotherapy, University of West Attica, Athens, Greece

**Keywords:** aging, cognition, dementia, inspiratory muscle training, respiratory muscle training

## Abstract

Cognitive decline and dementia represent a growing global health burden, particularly among older adults and populations with cardiopulmonary and vascular risk factors. While physical exercise has been shown to exert protective effects on cognition, the role of respiratory muscle training (RMT) remains unclear. The aim of this review was to investigate the effects of RMT on cognitive function and cognitive decline. Respiratory muscle training has been implemented in older adults with elevated blood pressure, post–COVID-19 patients, patients with chronic obstructive pulmonary disease (COPD), and patients with obstructive sleep apnea (OSA). There is only preliminary evidence regarding the effectiveness of inspiratory muscle training (IMT) on cognitive function, with only one study reporting statistically significant between-group differences (i.e., respiratory muscle training vs. control) in specific cognitive domains. Although respiratory muscle training appears to be a potentially promising intervention for improving cognitive function, the current evidence is limited. Further well-designed randomized controlled trials are required to draw definitive conclusions regarding its preventive role in cognitive decline and dementia.

## 1 Introduction

Cognitive impairment is characterized by deficits in memory, learning, attention, and decision-making that interfere with daily functioning. It is highly prevalent among older adults and commonly associated with neurodegenerative and vascular conditions, including Alzheimer's disease and Parkinson's disease (1). Given the projected growth of the global older adult population, which is expected to reach 1.4 billion by 2030, 2.1 billion by 2050, and 3.1 billion by 2,100, this demographic shift poses a substantial challenge for healthcare systems worldwide (2). The prevalence of mild cognitive impairment among adults aged over 60 years ranges from approximately 6.7%–25.2%, with a considerable proportion progressing to dementia. Cognitive decline significantly impairs instrumental activities of daily living, such as telephone use, transportation, and household management, often increasing the time and effort required for task completion (3). As cognitive decline progresses, individuals frequently experience reduced engagement in leisure activities, difficulty following cognitively demanding content, and challenges in maintaining personal hygiene (4).

Mild cognitive impairment may progress to dementia, and currently approved pharmacological treatments are limited to delaying disease progression in specific dementia subtypes. Consequently, preventive strategies aimed at delaying the onset of cognitive impairment are considered a critical approach. Proposed interventions include pharmacological agents, dietary supplements, physical activity and exercise, and cognitive training (1, 5). A recent systematic review and meta-analysis concluded that physical exercise—particularly moderate- to high-intensity aerobic exercise—can reduce global cognitive decline (6), with benefits primarily attributed to improvements in working memory. In addition, open-skill exercises (e.g., tennis, squash, boxing), which involve unpredictable environments and continuous decision-making, have been shown to elicit greater cognitive benefits than closed-skill exercises (e.g., running or cycling) (7).

Importantly, breathing-based interventions are mechanistically distinct from open-skill exercise and are generally classified as closed-skill activities. Nevertheless, breathing exercises may influence cognitive function through physiological and neurobiological pathways rather than task-related cognitive complexity. These effects have been attributed to activation of brain regions involved in cognition and memory, including cortical and limbic structures. A recent review examining stroke populations reported that breathing exercises may improve autonomic nervous system balance by enhancing parasympathetic activity, which is associated with favorable psychological and cognitive outcomes (8). Accordingly, beyond the cardiovascular benefits of aerobic exercise, respiratory function itself may represent an independent and complementary pathway influencing cognitive health. Therefore, the purpose of this narrative review was to investigate the effects of respiratory muscle training—a technique that augments respiratory function—on cognition and cognitive decline.

## 2 Respiratory function and cognitive decline

Lung function and respiratory diseases have been extensively studied as risk factors for dementia. Individuals with reduced lung function and those with respiratory disease—regardless of baseline lung function—particularly during midlife, appear to have an increased risk of dementia (9). These associations have been observed across different countries, research teams, and both sexes, even after adjustment for multiple confounders. Dementia has been linked to objective indicators of pulmonary dysfunction, including forced expiratory volume in one second, forced vital capacity, the FEV₁/FVC ratio, and clinically diagnosed COPD, asthma, and chronic bronchitis (10).

Several mechanisms may link reduced respiratory function to cognitive decline, including impaired cerebral oxygen delivery, prolonged low-grade hypoxia, and shared exposure to adverse environmental factors such as smoking, socioeconomic deprivation, and poor early-life nutrition (11). Prolonged hypoxia may also contribute to systemic inflammation, oxidative stress, sympathetic nervous system activation, reduced cerebral arterial elasticity, and microvascular damage (12).

In addition, reduced respiratory function may lead to lower levels of habitual physical activity, which is itself a well-established risk factor for cognitive decline. Conversely, by improving respiratory efficiency and reducing dyspnea, respiratory muscle training may facilitate greater engagement in physical activity, thereby indirectly supporting cognitive health. Given the multiple modulatory pathways linking respiration and cognition, improvements in respiratory function may enhance arterial oxygenation and cerebral oxygen delivery, supporting brain function (13). However, whether the cognitive effects of respiratory muscle training are direct or mediated through increased physical activity remains unclear.

## 3 Respiratory muscle training and cognition

The literature examining respiratory muscle training and cognitive outcomes is characterized by heterogeneous protocols, populations, and outcome measures, reflecting the complex and multifactorial nature of cognitive decline (Table 1).

**Table 1:** Description of selected studies included in the review.

Study	Population (n)	Intervention (RMT)	Control/comparator	Training parameters	Cognitive outcomes	Main results
Hamilton et al. (15)	Middle-aged and older adults with elevated systolic blood pressure (*n* = 30)	High-intensity inspiratory muscle training (IMT; *n* = 15)	Sham IMT (15% MIP; *n* = 15)	30 breaths/day, 6 days/week, 6 weeks; intensity 75% MIP	NIH toolbox cognition battery	Significant group × time interaction favoring IMT for episodic memory (*p* = 0.04) and processing speed (*p* = 0.02); improvement in fluid cognition composite score (*p* = 0.02)
Freeberg et al. (14)	Middle-aged and older adults (*n* = 16)	High-intensity IMT (75% MIP; *n* = 9)	Sham IMT (15% MIP; *n* = 7)	36 sessions over 6 weeks; progressive intensity 55%–75% MIP	NIH toolbox cognition battery	Within-group improvement in episodic memory in IMT group (*p* = 0.045); no significant group × time interaction for any cognitive domain
Cheng et al. (16)	Patients with COPD and mild cognitive impairment (MMSE 23–27; *n* = 48)	Combined IMT + EMT (RMT; *n* = 28)	IMT alone (*n* = 20)	30 breaths/session, twice daily, 7 days/week, 8 weeks; intensity 30% MIP/MEP with weekly 5% increases	MMSE	Within-group improvement in MMSE in RMT group (*p* < 0.05); no between-group difference (*p* = 0.58); *no true non-training control group*
del Corral et al. (17)	Post–COVID-19 patients (*n* = 88)	IMT (*n* = 22) or EMT (RMT; *n* = 22)	Sham IMT (*n* = 22) or sham EMT (*n* = 22)	40 min/day (2 × 20 min), 6 days/week, 8 weeks; intensity 20%–80% MIP/MEP	MoCA	No significant group × time interaction for cognitive outcomes; no between-group differences
Stavrou et al. (18)	Patients with obstructive sleep apnea (OSA; *n* = 28)	High-intensity RMT (IMT + EMT; *n* = 14)	CPAP therapy (*n* = 14)	3 sets of 12 repetitions/day, 4 weeks; intensity 80%–90% MIP/MEP	MoCA	Within-group improvement in total MoCA score (*p* < 0.001) and attention/working memory in RMT group; no significant between-group differences

RMT, respiratory muscle training; IMT, inspiratory muscle training; EMT, expiratory muscle training; MIP, maximal inspiratory pressure; MEP, maximal expiratory pressure; COPD, chronic obstructive pulmonary disease; OSA, obstructive sleep apnea; CPAP, continuous positive airway pressure; MoCA, Montreal cognitive assessment; MMSE, mini-mental state examination.

Two studies examined the effectiveness of IMT in middle-aged and older adults (14, 15). Freeberg et al. (14) enrolled 16 participants randomized to high-intensity IMT or sham training. The intervention included 36 sessions with intensity progressing from 55% to 75% of maximal inspiratory pressure, while sham training was performed at 15%. Hamilton et al. (15) examined middle-aged and older adults with above-normal systolic blood pressure assigned to a 6-week high-intensity IMT program or sham training.

Cheng et al. (16) investigated patients with COPD and mild cognitive impairment using IMT alone or combined inspiratory and expiratory muscle training (IMT + EMT; RMT). Del Corral et al. (17) studied post–COVID-19 patients using a four-arm, home-based respiratory muscle training protocol. Stavrou et al. (18) compared high-intensity respiratory muscle training with continuous positive airway pressure therapy in patients with OSA.

Across studies, intervention durations ranged from 4 to 8 weeks, with training frequencies typically being between to 6 and 7 days per week and intensities progressing from approximately 20%–30% to 75%–90% of maximal inspiratory or expiratory pressure.

Cognitive outcomes were assessed using heterogeneous tools, including the NIH Toolbox Cognition Battery, MMSE, and MoCA (19). Hamilton et al. (15) reported significant between-group improvements in episodic memory and processing speed, as well as in overall fluid cognition. In contrast, Freeberg et al. (14) observed a within-group improvement in episodic memory in the IMT group (*p* = 0.045) without a significant group × time interaction, indicating that this finding should be interpreted cautiously.

In Cheng et al. (16), no significant between-group differences were observed between IMT and RMT; however, a statistically significant within-group improvement was reported in the RMT group. This study lacked a non-training control group, which limits causal inference. Del Corral et al. (17) reported no significant between-group cognitive effects. Stavrou et al. (18) observed improvements in both intervention and control groups, with no statistically significant between-group differences, despite marked within-group gains in the RMT group.

## 4 Discussion

This review synthesizes preliminary evidence on the relationship between respiratory muscle training and cognitive outcomes. While cognitive decline is common among older adults, it is also influenced by modifiable cardiopulmonary and vascular risk factors (20). Elevated systolic blood pressure and COPD are both associated with cognitive impairment, potentially through systemic inflammation, hypoxia, and cerebrovascular dysfunction (21–23).

Mechanistic studies suggest that high-intensity IMT may influence autonomic regulation, cerebrovascular reactivity, and endothelial nitric oxide bioavailability (14, 24, 25). Neuroimaging studies further indicate that inspiratory loading activates cortical and subcortical networks involved in interoception, attention, and task engagement (26–28). However, the depth of mechanistic evidence currently exceeds the strength of the available clinical efficacy data, and these findings should be interpreted as hypothesis-generating rather than confirmatory.

Several methodological limitations must be acknowledged, including small sample sizes, short intervention durations, heterogeneous populations, variability in training protocols, and inconsistent cognitive outcome measures. Moreover, most studies reported within-group improvements without statistically significant between-group effects.

Accordingly, while respiratory muscle training may represent a feasible and low-cost adjunctive intervention, current evidence does not yet support definitive conclusions regarding its preventive efficacy against cognitive decline.

## 5 Conclusions

Respiratory muscle training may have beneficial effects on selected cognitive outcomes and holds promise as a supportive intervention in populations at increased risk of cognitive decline. However, the limited number of studies and substantial methodological heterogeneity preclude definitive conclusions. Future randomized controlled trials with adequate power, standardized cognitive assessments, and appropriate control conditions are required to clarify the role of respiratory muscle training in cognitive health.
